# Role of Intraoperative Pathology Consultation in Skeletal Tumors and Tumor-Like Lesions

**DOI:** 10.1155/2014/902104

**Published:** 2014-05-19

**Authors:** Poonam Bhaker, Harsh Mohan, Uma Handa, Sudhir Kumar

**Affiliations:** ^1^Department of Pathology, Research Block-A, Post Graduate Institute of Medical Education and Research, Sector-12, Chandigarh 160012, India; ^2^Department of Pathology, Sarai Building, Government Medical College and Hospital, Sector-32, Chandigarh 160030, India; ^3^Department of Orthopedics, Government Medical College and Hospital, Sector-32, Chandigarh 160030, India

## Abstract

Early and accurate detection of bone tumors and their staging are important since some of them are highly malignant. Intraoperative pathological consultation in bone tumors and tumor-like conditions is quite complex; however, it allows improvement in prognosis and limb salvage. Present study was conducted on 52 patients who underwent surgical procedure after clinical and radiological diagnosis of bone tumors/tumor-like conditions. Fresh unfixed tissue was quickly inspected grossly, followed by preparation of imprint smears and frozen section which were evaluated by two pathologists separately and compared subsequently with reports of paraffin-embedded sections. Clinical reasons for intraoperative consultation were to make diagnosis in 65.4% of cases and to determine resection margin status in 21.1% while in 13.5% of cases, it was for both indications. Diagnostic yield of imprint smears was 87.8% (13 malignant, 22 benign, and 1 tumor-like) and of frozen section was 90.2% (16 malignant, 19 benign, and 2 nonneoplastic) while paraffin sections could diagnose specific tumors in 95.1% (18 malignant, 18 benign, and 3 nonneoplastic). Although frozen section had better sensitivity (88.2%), it had less specificity (94.7%) as compared to imprint smears (76.5% and 100%, resp.). Imprint cytology and frozen section together provide a quick, safe, and reliable intraoperative provisional tissue diagnosis in skeletal tumors and tumor-like conditions.

## 1. Introduction

Bone tumors are infrequent but their detection is important since some of them are highly malignant. Incidence of sarcomas of the bone is about 8 to 9 per million population per year [1]. Clinically, the bone tumors may be simulated by infections, inflammatory conditions, or tumor-like conditions. Thus, it is critical to accurately diagnose and stage them because protocols for treating benign, malignant, or metastatic tumors are entirely different [2].

Currently, intraoperative consultation is considered as an integral part of surgical pathology and provides a rapid intraoperative diagnosis leading to timely and proper management of the patients [3]. In contrast to various other tissues, it has been less often utilized for bone lesions. This is because the primary bone tumors are uncommon and the characteristics of the material comprising these lesions render them difficult for quick sectioning [4]. It is tough to cut well mineralized tissue for frozen section; however, almost all malignant tumors of bone have soft areas that are suitable for frozen section analysis [5, 6].

Interpretation of intraoperative pathological consultation is a complex process that requires specialized histomorphologic knowledge which must be complemented by clinical, laboratory, and radiographic information [7]. Accurate intraoperative diagnosis may improve the patient's prognosis and success of salvage of the limb affected by the tumor. In view of limited studies on this subject in literature, present study was undertaken to evaluate the role of intraoperative pathological consultation (frozen section and tissue imprint cytology) in bone tumors and tumor-like lesions in making the diagnosis as well as in assessing the surgical margin status.

## 2. Materials and Methods

The present prospective study was conducted on 52 patients undergoing surgery (resection, biopsy, and/or resection limits) for bone tumors and tumor-like lesions from November 2009 to September 2011. For each case, detailed history, clinical, and radiological data were recorded. Fresh unfixed tissue specimen taken intraoperatively was wrapped in a wet gauze piece and quickly brought to tissue service laboratory of department of pathology. A careful and quick gross examination of the specimen was performed, followed by preparation of imprint smears and frozen sections.

Direct imprints were prepared by pressing the microglass slide gently against the freshly cut surface of specimen. Gliding movement was avoided. Air-dried and wet-fixed smears were prepared and stained by May Grünwald Giemsa (MGG) and hematoxylin and eosin (H&E), respectively [8]. After preparing imprint smears, the tissue was embedded in cryomatrix and quickly transferred to the cryostat (Shandon, UK) having a working temperature of −20°C. Sectioning (6-7 micron thick) was done and rapid H&E staining was performed [9].

Frozen section and imprint smears were evaluated simultaneously, but by independent observers. The results were communicated immediately to the operating surgeon verbally or in written format. Turnaround time between tissue accession and conveying of report was noted. The results of imprint smears and frozen section techniques were later compared with paraffin-embedded sections prepared from the resection specimen, which were considered as gold standard.

For statistical purpose, all malignant lesions were taken as positive and all benign lesions were taken as negative. Sensitivity, specificity, diagnostic accuracy, and total predictive value of each technique were calculated using descriptive statistics. Kappa test of agreement (*K*) was applied to observations of imprint cytology and frozen section to find the respective agreement with final histopathology report. *K* value more than 0.75 was considered to give excellent significance.

## 3. Results

Age of the patients ranged from 3 to 80 years with mean age of 26.6 ± 16.1 years. Male to female ratio was 1.6 : 1. Most frequent presenting feature was either pain (40.4%) or pain and swelling (36.6%). Most commonly affected bones were femur (34.6%), humerus (17.4%), tibia (11.5%), and sacrum (9.6%). Radiological diagnosis was benign in 22 cases (42.3%), malignant in 29 cases (55.7%), and inconclusive in 1 case (2.0%).

Clinical reasons for requesting an intraoperative pathological consultation were to make a diagnosis (34 cases, 65.4%) and to determine the adequacy of resection margins (11 cases, 21.1%), and in 7 cases (13.5%) it was required for both these purposes. For 18 cases, where the consultation was done for resection margin status, a total of 30 biopsies were evaluated. Previous tissue diagnosis was not available in majority of the cases (69.2%).

**Figure 1:**
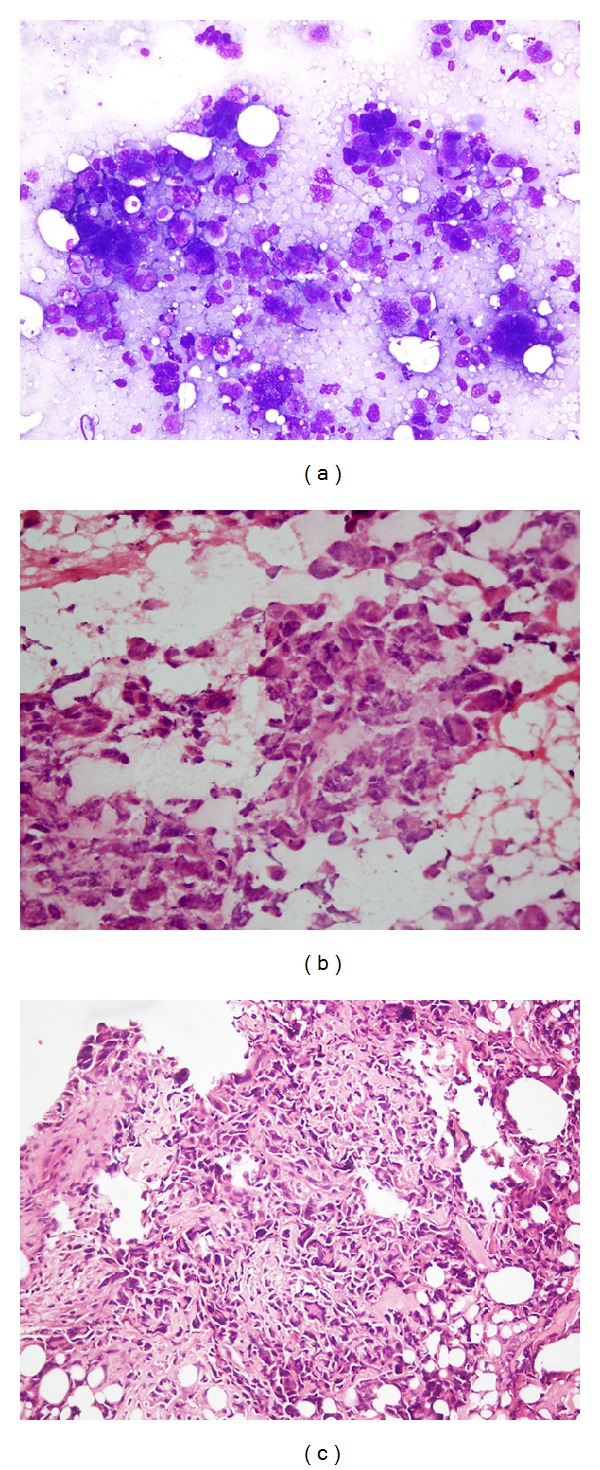
(a–c) Osteosarcoma: (a) highly pleomorphic and bizarre tumor cells on imprint smear (MGG stain, ×100); (b) frozen section showing highly anaplastic tumor cells with pale eosinophilic osteoid formation (H&E, ×400); (c) paraffin-embedded section showing pleomorphic and polymorphic tumor cells directly laying down osteoid (H&E, ×200).

**Figure 2:**
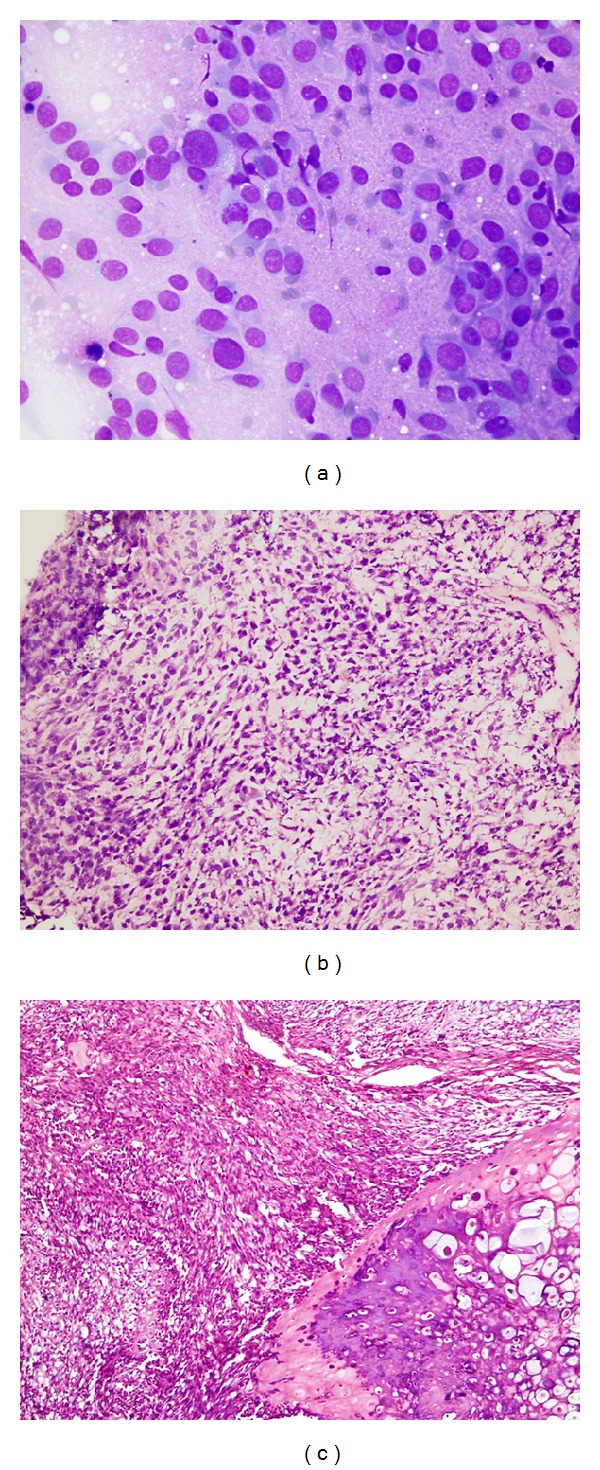
(a–c) Chondrosarcoma: (a) hypercellular smear showing singly scattered tumor cells having moderate pleomorphism and unipolar or bipolar cytoplasmic tags; an atypical mitotic figure is also present (MGG stain, ×400); (b) fascicles of tumor cells having moderate anaplasia which are noted in frozen section (H&E, ×200); (c) paraffin-embedded section displaying a tumor arranged in short fascicles with anaplastic spindle shaped cells. Right lower corner shows malignant chondroid differentiation (H&E, ×100).

**Figure 3:**
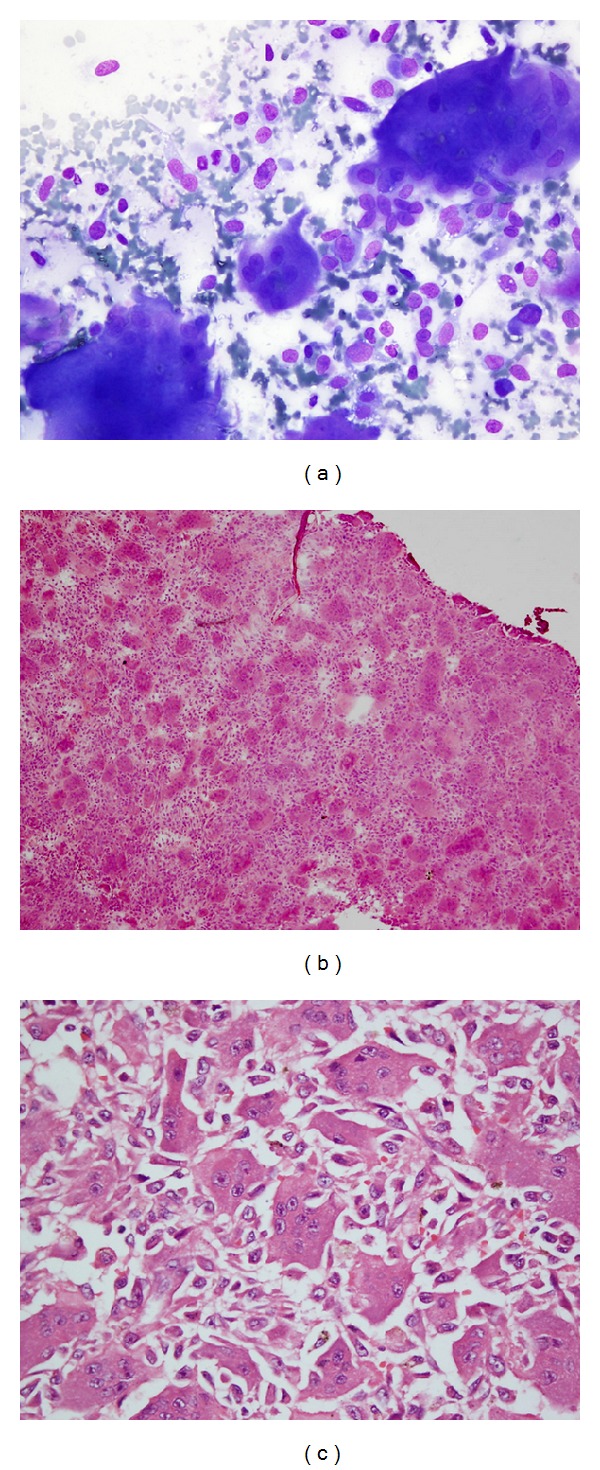
(a–c) Giant cell tumor: (a) multinucleate giant cells with nuclear morphology similar to that of background stromal cells in imprint smear (MGG stain, ×400); (b) uniformly distributed osteoclastic giant cells in a background of bland stromal cells noted in frozen section (H&E, ×100); (c) paraffin-embedded section confirming giant cell tumor (H&E, ×400).

**Figure 4:**
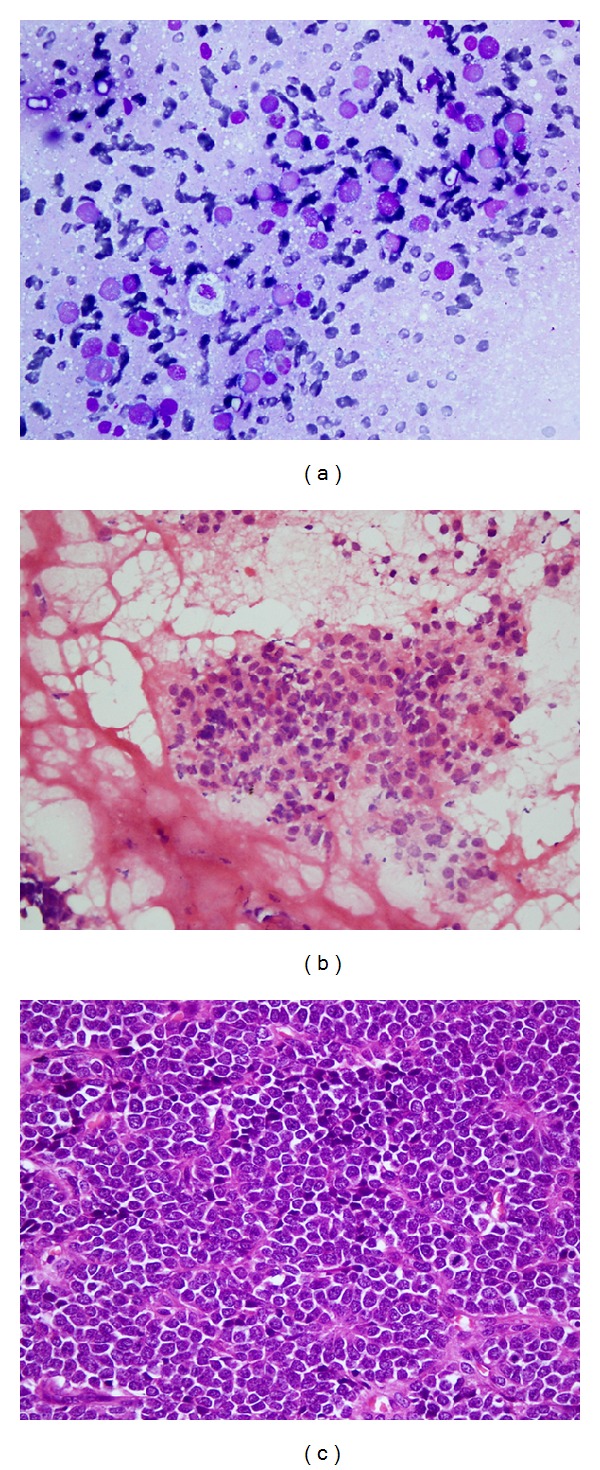
(a–c) Ewing's sarcoma: (a) imprint smear shows scattered population of malignant round tumor cells with larger cells having pale stained nuclei and smaller cells having darkly stained nuclei; cytoplasm is vacuolated (MGG stain, ×400); (b) frozen section shows sheet of small round tumor cells with occasional rosette formation (H&E, ×400); (c) malignant small round tumor cells arranged in sheets and pseudorosettes are identified in paraffin embedded section. Note mitotic figures (H&E, ×400).

Imprint smears were diagnostic in 87.8% cases (36 out of 41 cases) while the diagnostic yield of frozen section was 90.2% (37 out of 41 cases). The diagnostic yield of paraffin-embedded sections was 95.1% of cases (39 out of 41 cases). Among the 39 cases diagnosed on gold standard paraffin-embedded sections, there were 18 malignant neoplasms (46.15%), 18 benign neoplasms (46.15%), and 3 nonneoplastic lesions (7.7%). On imprint cytology, there were 13 malignant neoplasms (36.1%), 22 benign neoplasms (61.1%), and one nonneoplastic lesion (2.8%) while on frozen section, diagnosis of malignant neoplasm was made in 16 cases (43.2%), benign neoplasm in 19 cases (51.4%), and nonneoplastic lesion in 2 cases (5.4%). The diagnoses given by each technique and their correlation with paraffin-embedded sections are given in Table 1 and a few representative examples on tissue pathology are depicted in Figures 1, 2, 3, and 4. Among malignant neoplasms, Ewing's sarcoma and osteosarcoma were the most frequent while among benign neoplastic group giant cell tumor (GCT) was the most common.

The concordance rate between diagnoses of imprint cytology and paraffin-embedded sections was 88.9% (32 out of 36 cases). There were 4 false negative cases (11.1%) but no false positive result on imprint was seen. The concordance rate between diagnoses of frozen sections and paraffin-embedded sections was 91.7% (33 out of 36 cases). The discordance in 3 cases (8.3%) was due to one false positive case (2.77%) and 2 false negative cases (5.55%) on frozen section.

While assessing the adequacy of resection margin, imprint smears gave 2 false positive results and frozen section gave one false positive result. There was no false negative case with both techniques.

The statistical result of imprint smears and frozen sections for distinguishing malignant from benign lesions as well for determining the adequacy of resection margins is given in Table 2. The kappa test of agreement between imprint smear and paraffin-embedded section as well as between frozen section and paraffin-embedded section was highly significant.

The range of turnaround time for intraoperative pathological consultation was 5 to 35 minutes with average turnaround time of 18.7 minutes. In 75% of cases, turnaround time was less than or equal to 20 minutes.

## 4. Discussion

The diagnosis and management of primary musculoskeletal lesions are complex and associated with significant issues involving biopsy technique, treatment protocol, and postbiopsy management. The pitfalls can be overcome by utilizing a multidisciplinary approach to correlate clinical, radiological, and pathological information. Correct intraoperative histological assessment of bone lesions is crucial to select an appropriate surgical procedure and avoid under- and overtreatment of the patient.

Frequent reasons for requesting an intraoperative pathological consultation are to make or confirm a diagnosis, to determine the adequacy of resection margins, and to ascertain if the biopsy specimen is sufficient for making a definite diagnosis. Less commonly, it can also be employed to determine the extent of disease spread locally and beyond the local resection field, to assess an unsuspected finding at the time of operation, and to determine the presence or absence of residual or recurrent tumor after previous surgery [10]. Intraoperative pathological consultation can also be utilized to obtain fresh tissue for special studies [3]. In present study, the major reason for requesting intraoperative pathological consultation was to establish the diagnosis while it was demanded less frequently for assessing the adequacy of resection margins.

Intraoperative pathological consultation can be done by techniques such as frozen section and cytology (touch imprint, crush, or scrape), each having its own advantages and limitations. The diagnostic yield of imprint smears obtained in the present study (87.8%) parallels the diagnostic yield observed in study by Wisanuyotin et al. [4] while it is lower in comparison to that reported in another study from India [11]. The major reasons for inconclusive cytologic diagnosis in 5 cases in present study were hypocellular smears and nonrepresentative biopsy. The hypocellularity could be attributed to morphologic heterogeneity of tissue (such as fibrotic/sclerotic) or application of insufficient pressure while preparing the imprint slides.

The concordance rate between diagnoses of imprint cytology and paraffin-embedded sections in the present study was 88.9% which is lower compared to 94.9% reported in another study [11]. Sensitivity of imprint smears achieved in the present study was less in comparison with other studies while the specificity, positive predictive value, negative predictive value, and diagnostic accuracy compared well [4, 11]. The low sensitivity in our study was due to 11.1% false negative cases, which were mainly due to interpretive errors or nonrepresentative biopsy. One case was reported as benign chondroid lesion which turned out as well-differentiated chondrosarcoma on final histopathology. Another case reported as benign osteoblast-rich lesion on imprint smears was later diagnosed as low-grade osteosarcoma on paraffin sections. Two cases rendered as benign spindle cell lesion on imprint smear were subsequently diagnosed by paraffin sections as sarcoma NOS and biphasic malignant tumor. The reasons for misinterpretation in these cases were scanty cellularity or lack of pleomorphism, atypia, or mitosis in cases where cellular smears were obtained. It could be attributed to sampling error, where well-differentiated area of the lesion was biopsied; this problem was faced in diagnosing osteosarcoma and chondrosarcoma. Wilkerson and Crowell [12] have also reported difficulty in diagnosing chondrosarcoma by imprint smears owing to less number of cells and less prominent nuclear features of malignancy.

In terms of tumor typing, imprint smears correctly categorized all the malignant tumors into 4 major categories: malignant small round cell tumor, sarcoma, non-Hodgkin's lymphoma (NHL), and metastatic tumor. However, type specific diagnoses were possible to make in 76.9% of cases. The subtyping of sarcoma cases was difficult on imprint smears in the present study as the presence of osteoid or chondroid differentiation was not appreciated. Wisanuyotin et al. [4] too faced similar difficulties while providing provisional diagnosis in some specific malignancies on tissue imprints.

The diagnostic yield of frozen section was 90.2%; sampling errors were attributed to all 4 inconclusive cases. Almost similar diagnostic yield (93.4%) has been reported by Shah et al. [13]. Interpretative error accounted for single false positive case of aneurysmal bone cyst which was reported as borderline osteoblastic lesion on frozen section. The false negative cases were of osteosarcoma and chondrosarcoma, well-differentiated, which were diagnosed as benign tumor and chondromyxoid fibroma, respectively, on frozen sections. In both these cases, the cells did not display significant pleomorphism or cellular atypia. Diagnostic accuracy (91.66%) observed in the present study is close to that reported in other studies [13, 14]. However, a retrospective study has reported slightly higher sensitivity and specificity (100% and 91%, resp.) in making diagnosis [10].

Frozen section accurately typed 86.66% of malignant cases in terms of type of malignancy into 4 main categories: malignant small round cell tumor, sarcoma, NHL, and metastatic tumor. The sarcoma category could not be subtyped in all the cases as the respective differentiation was not recognized. In a study by Estrada-Villaseñor et al. [10], specific diagnosis was established in 86.3% of cases only and they found it difficult to diagnose osteosarcoma. Consistent results were seen in another study where frozen section made specific diagnosis in 85.9% of cases [13].

While determining the adequacy of resection margin, both imprint smears and frozen section techniques showed high diagnostic accuracy. Positive predictive value of imprint smears was quite low unlike that of frozen section. Surgical resection limits were revised in the cases reported as positive that is involved by the tumor. High negative predictive value by both techniques indicated that the surgery should be considered complete as the resection margins given negative by frozen section and imprint smears are expected to be negative on paraffin-embedded sections.

In the present study, better diagnostic yield was seen with frozen section compared to imprint smears. It was 2.4% more compared to imprint smears. However, difficulty was encountered while sectioning tissue from myxoid lesions. Paraffin-embedded section further improved diagnostic yield by 4.9%.

Frozen section was found to be superior to imprint smears and had better sensitivity but less specificity than imprint smears in making diagnosis. Frozen section diagnosed 7.1% more malignant cases in comparison to imprint cytology. False-negative results were less frequent on the frozen section compared to imprint evaluations. This may be due to better architectural details of frozen section. Imprint smears had advantages of being relatively inexpensive (without requirement of any special instrument) and less time consuming with rapid turnaround time. Imprints also gave better morphological details.

However, it is stated that attempt to make type specific intraoperative microscopic diagnosis of bone lesions is not necessary; distinguishing benign from primary malignant tumor and metastatic tumor is usually sufficient for correct management.

The turnaround time should be given due importance while providing intraoperative pathological consultation. Average turnaround time was less than 20 minutes. This was in accordance with observations of other workers [4, 7]. In the present study, the turnaround time exceeded 20 minutes in 25% of cases compared to 14% reported in another study [7]. The turnaround time was less with imprint cytology in comparison to frozen section, though it was not documented.

## 5. Conclusion

In conclusion, both frozen sections and imprint cytology provide a quick, safe, and reliable intraoperative provisional tissue diagnosis, especially to distinguish malignant from benign lesions. These techniques also provide reasonable assessment of adequacy of surgical resection limits, thus helping the surgeon to plan further treatment accordingly. Imprint cytology and frozen section complement each other in ensuring rapid tissue diagnosis. Intraoperative pathological consultation (frozen section and imprint cytology together) proves to be a valuable addition to the armamentarium in evaluating musculoskeletal tumors.

## Figures and Tables

**Table 1:** Correlation of frozen section and imprint cytology with paraffin-embedded sections*.

Paraffin-embedded section (*n* = 39)	Frozen section (*n* = 39)	Imprint smear (*n* = 39)
Osteosarcoma (*n* = 5)	Osteosarcoma (*n* = 2) Malignant tumor, typing not possible (*n* = 1) Sarcoma NOS (*n* = 1) Benign tumor (*n* = 1)	Osteosarcoma (*n* = 1) Sarcoma NOS (*n* = 2) Benign osteoblastic tumor (*n* = 1) Inconclusive (*n* = 1)

Chondrosarcoma, well differentiated (*n* = 1) Chondrosarcoma, dedifferentiated (*n* = 1)	Chondromyxoid fibroma (*n* = 1) Sarcoma NOS (*n* = 1)	Benign chondroid lesion (*n* = 1) Sarcoma NOS (*n* = 1)

Ewing's sarcoma (*n* = 5)	Ewing's sarcoma (*n* = 1) Malignant small round cell tumor (*n* = 3) Sarcoma NOS (*n* = 1)	Ewing's sarcoma (*n* = 2) Malignant small round cell tumor (*n* = 3)

Non-Hodgkin's lymphoma (*n* = 2)	Lymphoma (*n* = 1) Malignant undifferentiated tumor (*n* = 1)	Lymphoma (*n* = 1) Malignant small round cell tumor (*n* = 1)

Biphasic tumor, possibilities: adamantinoma and synovial sarcoma (*n* = 1)	Malignant spindle cell tumor (*n* = 1)	Benign spindle cell tumor (*n* = 1)

Sarcoma NOS (*n* = 1)	Inconclusive (*n* = 1)	Benign spindle cell tumor (*n* = 1)

Malignant undifferentiated tumor (*n* = 1)	Sarcoma NOS (*n* = 1)	Sarcoma NOS (*n* = 1)

Metastatic carcinomatous deposits (*n* = 1)	Metastatic carcinomatous deposits (*n* = 1)	Metastatic carcinomatous deposits (*n* = 1)

Giant cell tumor (*n* = 10)	Giant cell tumor (*n* = 9) Giant cell rich benign lesion (*n* = 1)	Giant cell tumor (*n* = 9) Giant cell rich benign lesion (*n* = 1)

Chondromyxoid fibroma (*n* = 3)	Chondromyxoid fibroma (*n* = 1) Chondroid lesion, possibly chondroblastoma (*n* = 1) Giant cell tumor (*n* = 1)	Giant cell rich benign lesion (*n* = 3)

Ossifying fibroma (*n* = 2)	Inconclusive (*n* = 2)	Giant cell rich benign lesion (*n* = 1) Inconclusive (*n* = 1)

Benign fibrous histiocytoma (*n* = 1)	Benign spindle cell lesion (*n* = 1)	Benign spindle cell lesion (*n* = 1)

Osteochondroma (*n* = 1)	Benign osteocartilaginous lesion (*n* = 1)	Giant cell rich benign lesion (*n* = 1)

Enchondroma (*n* = 1)	Benign chondroid lesion (*n* = 1)	Chondroma (*n* = 1)

Aneurysmal bone cyst (*n* = 2)	Aneurysmal bone cyst (*n* = 1) Borderline osteoblastic lesion (*n* = 1)	Giant cell rich lesion (*n* = 1) Benign cystic lesion (*n* = 1)

Unicameral bone cyst (*n* = 1)	Benign cystic lesion (*n* = 1)	Benign cystic lesion (*n* = 1)

*Two cases inconclusive on final sections were excluded.

**Table 2:** Statistical analyses of results given by imprint smear and frozen section technique.

	Imprint smear		Frozen section	
	Diagnosis	Resection margin	Diagnosis	Resection margin
Sensitivity (%)	76.47	100	88.2	100
Specificity (%)	100	92.3	94.7	96.15
Positive predictive value (%)	100	66.66	93.75	80
Negative predictive value (%)	82.6	100	90	100
Diagnostic adequacy (%)	88.88	93.3	91.66	96.66
Kappa test of agreement	0.77	0.76	0.83	0.86
